# Attraction of mosquitoes to primate odours and implications for zoonotic *Plasmodium* transmission

**DOI:** 10.1111/mve.12402

**Published:** 2019-08-17

**Authors:** J. W. Bakker, D. E. Loy, W. Takken, B. H. Hahn, N. O. Verhulst

**Affiliations:** ^1^ Laboratory of Entomology Wageningen University & Research Wageningen The Netherlands; ^2^ Departments of Medicine and Microbiology, Perelman School of Medicine University of Pennsylvania Philadelphia PA U.S.A.; ^3^ National Centre for Vector Entomology, Institute of Parasitology, Vetsuisse Faculty University of Zurich Zurich Switzerland

**Keywords:** *Anopheles*, *Plasmodium*, chimpanzee, Congo, bridge vectors, mosquito host preference, transmission dynamics

## Abstract

Vector‐borne diseases often originate from wildlife and can spill over into the human population. One of the most important determinants of vector‐borne disease transmission is the host preference of mosquitoes. Mosquitoes with a specialised host preference are guided by body odours to find their hosts in addition to carbon dioxide. Little is known about the role of mosquito host preference in the spillover of pathogenic agents from humans towards animals and vice versa. In the Republic of Congo, the attraction of mosquitoes to primate host odours was determined, as well as their possible role as malaria vectors, using odour‐baited traps mimicking the potential hosts of mosquitoes. Most of the mosquito species caught showed a generalistic host preference. *Anopheles obscurus* was the most abundant *Anopheles* mosquito, with a generalistic host preference observed from the olfactory response and the detection of various *Plasmodium* parasites. Interestingly, *Culex decens* showed a much higher attraction towards chimpanzee odours than to human or cow odours. Human *Plasmodium* parasites were observed in both human and chimpanzee blood, although not in the *Anopheles* mosquitoes that were collected. Understanding the role of mosquito host preference for cross‐species parasite transmission provides information that will help to determine the risk of spillover of vector‐borne diseases.

## Graphical abstract

- The majority of the mosquito species caught showed a generalistic host preference.
- *Anopheles obscurus* was the most abundant *Anopheles* mosquito, with a generalistic host preference, whereas *Culex decens* showed a much higher attraction towards chimpanzee odours than to human or cow odours
- Human *Plasmodium* parasites were observed in both human and chimpanzee blood, although not in the *Anopheles* mosquitoes that were collected.

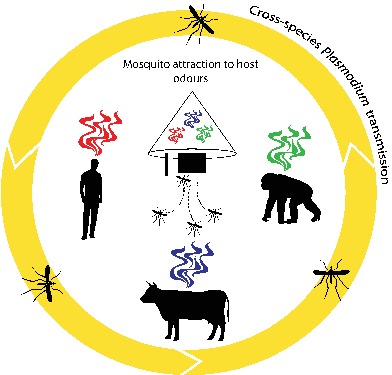

## Introduction

Many of the most deadly human diseases, including malaria, Zika virus, dengue virus and chikungunya virus, are transmitted by mosquitoes (Takken & Knols 1999; Harrington *et al*., 2001; Lambrechts *et al*., 2010; Fauci and Morens, 2016). In addition to transmitting diseases between human hosts, mosquitoes also facilitate the spillover of zoonotic pathogens into human populations (Jones *et al*., 2008). As the closest relatives of *Homo sapiens*, great apes are of interest as potential reservoirs of zoonotic vector‐borne pathogens themselves, and mosquitoes may act as a bridge vector between great ape reservoirs of such pathogens and humans. There is, however, a fundamental gap in knowledge concerning the transmission dynamics of vector‐borne diseases between great apes and humans, as well as the potential zoonotic threat that they pose to humans.

Studies of *Plasmodium* parasites in African great apes revealed that the malaria parasites *Plasmodium falciparum* and *Plasmodium vivax*, which together account for the majority of global malaria cases (WHO World Malaria Report 2018), both originated from parasites that infect African great apes (Liu *et al*., 2010, 2014; Loy *et al*., 2018a). Indeed, African great apes are infected with at least 13 *Plasmodium* species, which are further subdivided into the *Laverania* and *Plasmodium* subgenera (Liu *et al*., 2010, 2014, 2016; Loy *et al*., 2016,2017). The *Laverania* parasites tend to be host restricted, whereas *Plasmodium* parasites appear to have a more promiscuous host tropism.

Although several studies have failed to detect evidence of ape *Plasmodium* parasites in modern African humans ( Sundararaman *et al*., 2013; Délicat‐Loembet *et al*., 2015; Loy *et al*., 2018b), it is not clear whether this is representive of biological barriers to infection or a lack of exposure to ape parasites. Interestingly, studies of sanctuary apes show that the *Laverania* host‐species restriction observed in the wild can be broken when chimpanzees and gorillas are housed in the same sanctuary (Ngoubangoye *et al*., 2016), suggesting that perhaps ecological factors (such as the frequency of exposure to infectious mosquitoes) impact cross‐species transmission.

Although two studies have identified some of the *Anopheles* species that transmit ape *Plasmodium* parasites (Paupy *et al*., 2013; Makanga *et al*., 2016), little is known about the behaviour of mosquitoes that could serve as bridge vectors between ape and human hosts. One understudied area is the biting behaviour and host species preferences of the mosquitoes that are found in the forest near *Plasmodium‐*infected apes. Biting behaviour is largely dependent on the mosquito's host preference, which in turn is influenced by the body odour profile of the vertebrate hosts (Takken & Verhulst 2017). Some mosquito species have a specialised attraction towards humans (anthropophilic species), whereas others have a more opportunistic host preference (generalistic species) (Busula *et al*., 2015). This preference is mediated by differences in volatile compounds produced by different host species (Busula *et al*., 2017) and similarities in the odour profile of host species could mediate the transmission of pathogens between these species (Verhulst *et al*., 2012; Verhulst *et al*., 2018).

In the present study, experiments were performed at the Tchimpounga Chimpanzee Rehabilitation Centre in the Republic of Congo, where chimpanzees were previously reported to harbour ape *Plasmodium* parasites (Pacheco *et al*., 2013), aiming to examine the feeding behaviour and host choice of local mosquito species. Mosquito traps were baited with different host volatiles to assess the behaviour of mosquitoes towards different host odours. The present study also aimed to characterize the transmission dynamics of ape *Plasmodium* parasites by screening chimpanzees and mosquitoes for *Plasmodium* species.

## Materials and methods

### *Location*

Mosquito and chimpanzee blood samples were collected at the Jane Goodall Institute (JGI) Tchimpounga Chimpanzee Rehabilitation Centre (TC) in the Tchimpounga National Reserve, Republic of Congo (4°24′S, 11°48′E). Chimpanzee blood and faecal samples were collected by the veterinary staff of TC from October to November 2015 and October to January 2016, respectively. Mosquito samples were collected from October 2015 to January 2016 at three small islands within the TC that act as natural enclosures. The islands are located in the Kouilou river and house a dormitory where chimpanzees sleep during the night. The chimpanzees can roam free over the islands during the day. Sample collection was approved by the Ministry of Forest Economy and Sustainable Development of the Republic of Congo under permit No. 071. Chimpanzee blood samples were exported by the JGI TC under the Republic of Congo Cites export Permit No. 007008 and 010 (26 February 2016) and imported by the University of Pennsylvania under U.S. Cites Import Permit No. 15US7151B/9 and PHS Import Permit No. 2015‐10‐089. Mosquitoes were imported by the University of Pennsylvania under U.S. PHS Import Permit No. 2016‐02‐084.

### *Mosquito trapping*

Using odour‐baited mosquito traps, the host preference and species composition of a population of mosquitoes can be determined (Qiu *et al*., 2007). Two different odour‐baited traps were used in the present study: the BG‐Sentinel trap (BioGents GmbH, Regensburg, Germany) and the Suna trap ( BioGents GmbH, Regensburg, Germany). Carbon dioxide(CO_2_) was supplemented to each trap because it is a general long‐distance mosquito activator and attractant (Schmied *et al*., 2008; Smallegange *et al*., 2010).

To test for the most appropriate mosquito trap for trapping *Anopheles* mosquitoes in the Tchimpounga National Reserve, the BG‐Sentinel trap was compared with the Suna trap. The traps were baited with a standardized five‐component odour blend (Menger *et al*., 2014; Pombi *et al*., 2014) and CO_2_ to attract mosquitoes (Smallegange *et al*., 2010). Three traps of each type were placed along a transect 30 m apart with similar spatial conditions and rotated using a complete randomized design resulting in six trapping nights.

Carbon dioxide was produced using sugar‐fermenting yeast as an organic source of CO_2_. Cane sugar and molasses were both used as sugar source. Carbon dioxide was produced using 125 g of cane sugar or molasses, 9 g of yeast and 1 L of water in a 1.5‐L bottle (Mweresa *et al*., 2014). Additionally, to increase CO_2_ production, CO_2_ was produced using 250 g of cane sugar or molasses with 17 g of yeast and 2 L of water in a 5‐L bottle (Mweresa *et al*., 2014). Water from the Kouilou River was used during field experiments. The CO_2_ was released using 70 cm of silicon tubing inserted in the cap of the bottle and connected to the mosquito trap.

Host odours from chimpanzees, cows and humans were collected using nylon socks (20 DEN, 100X polyamide; HEMA, De Bilt, The Netherlands). Cow and chimpanzee odours were collected by rubbing a nylon sock on the arm (for chimpanzees) or upper leg (for cows) of the animal for 30 s. Human odour was collected from local male individuals wearing a nylon sock overnight. To minimize the influence of anthropogenic compounds, these individuals did not use deodorant or any perfumed substances the day before wearing a nylon sock overnight. Animal and human odours were collected from three different individuals. The socks were cut into three pieces and one piece of each individual were combined. To determine the most suitable *Anopheles* trap, a synthetic blend mimicking human odour containing ammonia, (*S*)‐lactic acid, tetradecanoic acid, 3‐methyl‐1‐butanol and butan‐1‐amine (Menger *et al*. 2014) was released from the two different trap models, Suna and BG‐sentinel. A clean nylon sock was used as a control. All nylon socks were handled with clean latex gloves and stored in glass jars.

The mosquito traps were operated from 17.00 h to 06.30 h. Suna traps were hung with the trap entry located 30 cm above ground level (Hiscox *et al*., 2014), whereas the BG‐Sentinel trap was placed on the ground (Schmied *et al*., 2008). Latex gloves were worn when handling the traps to avoid contamination with odours and the traps were cleaned with 70% ethanol after each trapping night (Busula *et al*., 2015).

### *Odour preference of different mosquito species*

To assess the attractiveness of mosquitoes to natural host odours, five odour‐baited Suna traps were baited with five different odour treatments consisting of (a) sock with no odour and no CO_2_; (b) sock with no odour with CO_2_; (c) sock with cow odour and CO_2_; (d) sock with chimpanzee odour and CO_2_; and (v) sock with human odour and CO_2_. Each trap was set at least 30 m apart and odour baits randomized in a 5 × 5 Latin square experimental design that was repeated seven times. Location one was 4 m in front of a chimpanzee dormitory housing 23 chimpanzees; location two was behind a house where three to four caregivers were constantly present, as well as close (15 m) to the chimpanzee dormitory; location three was near a small settlement of wooden houses with humans; location four was at the edge of a forest, 10 m from wooden houses occupied by humans; and location five was in the forest with no humans and chimpanzees within a 60 m range.

### *Chimpanzee blood sampling*

Blood samples were collected from 84 chimpanzees (*Pan troglodytes*) living at the Tchimpounga Chimpanzee Rehabilitation Centre. Samples were collected in ethylenediamine tetraacetic acid collection tubes after routine veterinary examination. Density gradient centrifugation was used to separate red blood cells (RBC). To concentrate RBCs, 2–3 mL of whole blood was diluted in phosphate‐buffered saline (1:1 v/v) and gently placed on one volume of Lymphoprep (Axis‐Shield, Oslo, Norway). The mixture was then centrifuged at approximately 700 rpm for 35 min. After removal of the plasma, the RBC were mixed with RNAlater® (1:1 v/v) (Thermo Fisher, Waltham, MA, U.S.A.) in a 15‐mL tube. The plasma was mixed with 1:1 (v/v) RNAlater® in a 15‐mL tube. Blood samples were stored at −20 °C before shipment, shipped at ambient temperature and stored at −80 °C upon arrival. DNA was extracted from chimpanzee blood samples using the DNeasy Blood and Tissue kit (Qiagen, Valencia, CA, U.S.A.).

### *Mosquito identification*

Mosquitoes collected from the traps were killed using ethyl acetate (non‐acetonic nail polish, Kruidvat, The Netherlands) and morphologically identified to genus level using taxonomic keys (Highton, 1983). *Anopheles* mosquitoes were identified to species level (Gillies and Coetzee, 1987). After morphological identification, *Anopheles* mosquitoes were stored in 200 μL tubes with RNAlater® (Ambion, Austin, TX, U.S.A.) and stored at −20 °C. Non‐*Anopheles* mosquitoes were placed in 1.5‐mL Eppendorf tubes with silica beads (Sigma‐Aldrich, St Louis, MO, U.S.A.) and stored at −20 °C. *Anopheles* mosquitoes identified as part of a species complex were further identified by polymerase chain reaction (PCR) amplification of the *Cytochrome oxidase subunit II* (COII) gene as described previously by Ndo *et al*. (2010). Positive PCR products for COII were purified and sequenced using Sanger sequencing. Blood meals from culicine mosquitoes (*n* = 244) were preserved on filter paper cards (Whatman FTA cards; GE Heathcare, Chicago, IL, U.S.A.). The blood meals were spread onto FTA cards by pressing the blood out of the abdomen using a sterile pipet tip. The FTA cards with blood spots were dried for 1–2 h at room temperature and stored in a CloneSaver pouch (GE Healthcare) together with silica gel (Sigma‐Aldrich) for 1–3 months. Mosquitoes and FTA cards were shipped at ambient temperature and stored at −80 °C before nucleic acid extraction.

#### DNA extraction

DNA was extracted from *Anopheles* mosquitoes using the DNeasy 96 Blood & Tissue Kit (Qiagen) in accordance with the manufacturer's instructions for DNA purification from insects. Briefly, mosquitoes were placed in 1.5‐mL Eppendorf tubes containing 180 μL of Buffer ATL (Qiagen) and 20 μL of proteinase K (Qiagen) and crushed using a 1‐mL pipette tip or mortar. Samples were incubated at 56 °C overnight in an incubator. DNA was further extracted in accordance with the manufacturer's instructions. DNA was extracted from 1900 individual mosquitoes and the remainder (3500 mosquitoes) were pooled (five mosquitoes per pool) to reduce the cost of DNA extraction. After DNA extraction, 50 μL of the DNA extract was treated with a One Step™ PCR inhibitor removal kit (Zymo Research, Irvine, CA, U.S.A.) before PCR amplification.

DNA was extracted from CloneSaver FTA filter paper cards (GE Healthcare) using the Allprep DNA/RNA mini kit (Qiagen). Briefly, two or three disks of the dried blood spots were punched out using a Harris 3‐mm micro‐puncher (GE Healthcare). Two or three discs were mixed in a 1,5‐mL Eppendorf tube with 350 μL of RLT buffer (Qiagen) containing 1% β‐mercaptoethanol and incubated for 1 h at 37 °C with shaking (1000 rpm). Afterwards, DNA/RNA was extracted in accordance with the manufacturer's instructions.

#### *Plasmodium* parasite screening

All blood and mosquito samples were screened for presence of *Plasmodium* DNA using pan‐*Plasmodium* primers targeting the mitochondrial cytochrome *b* gene (*cytB*) (956 bp) by nested PCR as described previously (Liu *et al*., 2010). Amplicons were sequenced directly without interim cloning using Sanger sequencing technology. All obtained mosquito positive sequences were aligned with GenBank reference sequences (see Supporting information, Table S1) using clustalw, version 2.1 (http://www.clustal.org) in geneious, version 11 (https://www.geneious.com). jmodeltest, version 2.1.7 (Darriba *et al*., 2012) was used to select for best evolutionary model. Maximum likelihood phylogenies with bootstrap support (1000 replicates) were estimated using phyml, version 3 (http://www.atgc-montpellier.fr/phyml) and GTR + G + I as evolutionary models (Guindon *et al*., 2010).

### *Nucleotide sequence accession numbers*

All newly derived mosquito *Plasmodiidae* sequences have been submitted to GenBank with accession numbers MK502145 to MK502166.

### *Statistical analysis*

A generalized linear model (GLM) with binomial distribution and logit link function was used to test the difference in trapping efficacy of the BG‐Sentinel trap and the Suna trap, as well as the attractiveness of different host odours to different mosquito species. Differences in treatments were expressed as the number of mosquitoes caught (per species) in one trap divided by the total number of mosquitoes (per species) trapped during each trapping night (Busula *et al*., 2015). Effects of location, day, temperature, humidity, CO_2_ treatment and mosquito species, as well as their two‐way interactions, on the number of mosquitoes caught were fitted in the GLM and non‐significant factors were removed. Models were compared by the corrected Akaike's information criterion. *P* < 0.05 was considered statistically significant. All statistical analyses were performed using spss, version 20 (IBM Corp., Armonk, NY, U.S.A.).

## Results

### *Mosquito host preference and identification*

#### Mosquito trapping

In total, 5145 *Anopheles* mosquitoes were caught during the study period, the vast majority of which (*n* = 5002) were identified as *Anopheles obscurus*. The remaining *Anopheles* mosquitoes were identified as *Anopheles paludis*, *Anopheles moucheti*, *Anopheles ziemanni*, *Anopheles gambiae s.l*. and *Anopheles nili* (see Supporting information, Table S2)*. Anopheles moucheti* (*n* = 4), *An. nili* (*n* = 6) and *An. gambiae s.l*. (*n* = 21) are known vectors of human *Plasmodia* spp. (Wondji *et al*., 2002; Cohuet *et al*., 2004; Sinka *et al*., 2012) and represented 0.6% of all anophelines caught in the present study. Other mosquito species consisted of 6097 *Mansonia africana*, 1742 *Culex* spp., 549 *Coquillettidia* spp. and 68 *Aedes* spp.

**Figure 1:**
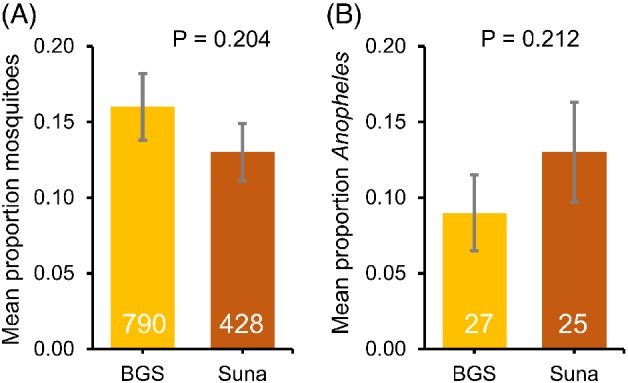
Trapping efficiency of two odour‐baited mosquito traps. Back‐transformed mean proportion [generalized linear model (GLM)] of caught mosquitoes per trap using the BG‐Sentinel (BGS) trap (*n* = 15) and the Suna trap (*n* = 15) baited with the five‐component odour blend odour blend (Menger *et al*., 2014) and CO_2_. Numbers in the bars indicate the total number of mosquito spp. (A) and *Anopheles* spp. (B) trapped. Error bars represent the SEM. Location effect was significant for both total mosquito spp. and total *Anopheles* spp. and included in the GLM (*P* < 0.001). [Colour figure can be viewed at http://wileyonlinelibrary.com].

The BG‐Sentinel trap caught more mosquitoes per trap (mean ± SE: 43.89 ± 10.34) than the Suna trap (25.17 ± 6.11); however, these means were not significantly different after inclusion of the location effect in the GLM (GLM, *P* = 0.204 for trap and *P* < 0.001 for location) (Fig. 1). The mean number of *Anopheles* mosquitoes per trap per day was 1.50 ± 0.59 for the BG‐Sentinel trap and 1.47 ± 0.5 for the Suna trap. Because the mosquito trap catches were not significantly different between the two traps and the Suna trap is more weather resistant than the BG‐Sentinel trap, the Suna trap was chosen for further experiments.

#### Host preference of different mosquito species

The majority of the species caught during 35 trapping nights were *Anopheles* spp. (*n* = 3347), *Mansonia* spp. (*n* = 3542), *Culex* spp. (*n* = 1645) and *Coquillettidia* spp. (*n* = 541).

Mosquito catches were significantly affected by trap location (GLM, *P* < 0.001) and odour treatments (*P* < 0.001). In addition, there was a significant interaction between the type of odour bait used and mosquito species (GLM, *P* < 0.038), indicating that the odour baits differentially attracted the mosquito species present in the area. At some locations, certain mosquito species were more abundant than others, as indicated by an interaction between mosquito species and location in the GLM (*P* < 0.001). The significant interactions between mosquito species, odour and location indicated that different mosquito species respond differently to a variation of odours and are more abundant at different locations.

**Figure 2:**
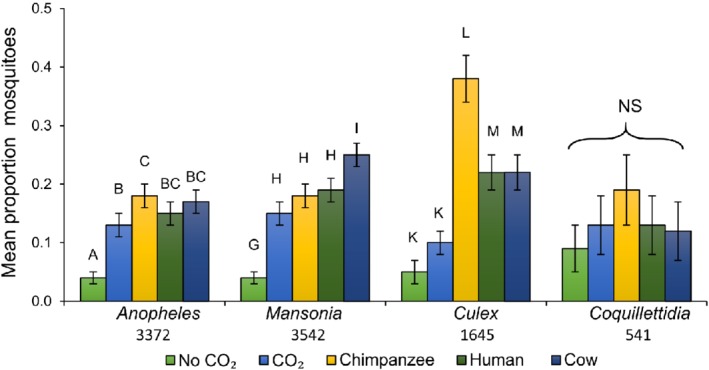
Attraction of mosquito species to different host odours trapped with odour baited Suna traps. Back‐transformed mean proportion [generalized linear model (GLM)] of mosquito genera caught using different host odour treatments: no odours, CO_2_ only, chimpanzee odours with CO_2_, human odours with CO_2_ and cow odours with CO_2_. Error bars represent the SEM. Numbers below the bars indicate the total number of mosquitoes caught per species (*n* = 35 trapping nights). Different uppercase letters indicate significant differences between odour baits within each genus (GLM with least significant difference post‐hoc test, *P* < 0.05). NS, non‐significant (*P* > 0.05). [Colour figure can be viewed at http://wileyonlinelibrary.com].

Significantly more *Anopheles* mosquitoes were caught in the presence of chimpanzee, human, or cow odours compared with traps baited with CO_2_ alone or without any bait (*P* < 0.001, GLM) (Fig. 2 and Table 1; see also Supporting information, Table S3 and Figure S1). However, *Anopheles* mosquitoes (97% of which were identified as *An. obscurus*) did not show a specific host preference for chimpanzee (mean 29.9 ± 7.6), cow (28.3 ± 8.9) or human (27.0 ± 9.4) odours (*P* > 0.05, GLM) (Fig. 2 and Table 1; see also Supporting information, Table S3 and Figure S1).

**Table 1:** Mean number of trapped mosquitoes per night per odour bait.

	Odour bait (mean ± SE)				
Mosquito species	No CO_2_	CO_2_	Chimpanzee + CO_2_	Human + CO_2_	Cow + CO_2_
*Anopheles*	5.7 ± 2.7^a^	21.6 ± 7.0^b^	29.9 ± 7.6^c^	27.0 ± 9.4^bc^	28.3 ± 8.9^bc^
*Mansonia*	10.4 ± 3.0^g^	20.9 ± 6.5^h^	25.1 ± 4.7^h^	27.6 ± 8.5^h^	34.1 ± 8.5^i^
*Culex*	3.1 ± 1.4^k^	6.5 ± 1.5^k^	20.4 ± 4.9^l^	12.8 ± 3.6^m^	12.0 ± 2.4^m^
*Coquillettidia*	2.1 ± 0.8	2.8 ± 1.0	5.0 ± 1.6	4.2 ± 2.3	3.9 ± 1.2

There were 35 trapping nights in total. Different superscript lowercase letters indicate significant differences between odour baits for each species (generalized linear model with least significant difference post‐hoc test, *P* < 0.05). No significant differences were found for *Coquillettidia*.

The *Mansonia* species caught during the present study were morphologically identified as *Mansonia africana*. Unlike *An. obscurus*, *M. africana* exhibited a preference for cow odour (*P* < 0.05, GLM) (Fig. 2 and Table 1; see also Supporting information, Table S3). Traps baited with cow odour caught an average of 34.1 ± 8.5 *M. africana*, which was more than three times higher than traps with no bait (10.4 ± 3.0, *P* < 0.001, GLM). No significant differences were found between chimpanzee or human odours (25.1 ± 4.7, 27.6 ± 8.5, respectively). Traps without bait attracted significantly fewer *Mansonia* species than with bait (*P* < 0.05, GLM) (Fig. 2 and Table 1; see also Supporting information, Table S3 and Figure S1).

The *Culex* species caught in the present study were morphologically identified as *Culex decens*. Traps baited with chimpanzee odour caught approximately two‐fold more *Culex decens* (20.4 ± 4.9) than traps baited with cow (12.0 ± 2.4, *P* = 0.002, GLM) or human odour (12.8 ± 3.6, *P* = 0.002, GLM). Adding CO_2_ to a Suna trap did not result in a significant increase in mosquito catches compared with traps with no bait (*P* = 0.083, GLM) (Fig. 2 and Table 1).

No significant differences in mosquito attraction towards different host odours were observed for *Coquillettidia* species (*n* = 541, *P* > 0.05) (Fig. 2 and Table 1; see also Supporting information, Table S3 and Figure S1).

#### *Plasmodium* detection

**Figure 3:**
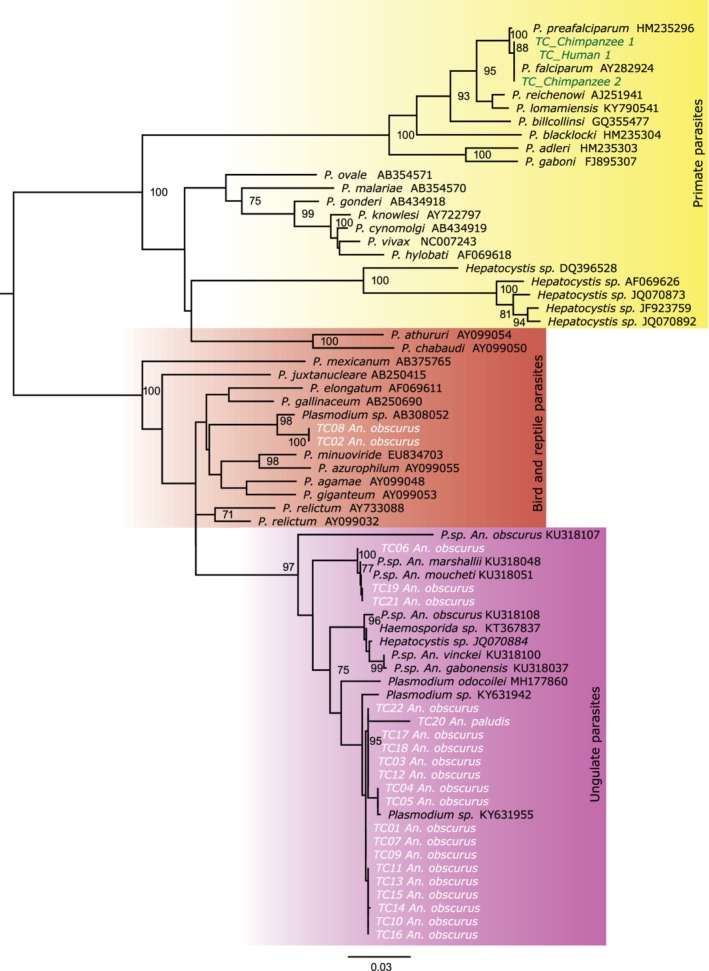
Evolutionary relationships of *Plasmodium* parasite sequences from chimpanzees and humans. A maximum likelihood tree of mitochondrial cytochrome *b* (*cytB*) sequences (956 bp) is shown. Sequences in green represent the *Plasmodium* sequences found in chimpanzee (Chimpanzee 1 and 2) and human (Human 1) blood samples. Sequences in white represent *Plasmodiidae* sequences obtained from *Anopheles* mosquitoes. Black sequences represent *Plasmodium* reference sequences. Coloured blocks indicate primate (yellow), bird and reptile (brown), and ungulate (purple) reference sequences. Bootstrap values (≥ 70%) are shown above and below the branch nodes. The scale bar represents 0.03 nucleotide substitutions per site. [Colour figure can be viewed at http://wileyonlinelibrary.com].

In total, 84 chimpanzees (*Pan troglodytes*) were screened for *Plasmodium* species. Despite the fact that other groups have detected ape *Plasmodium* species at this field site (Pacheco *et al*., 2013), no ape *Plasmodium* species were detected during the course of the present study. However, two chimpanzees were found to be infected with the human parasite *P. falciparum* (Fig. 3).

The *Anopheles* mosquitoes (*n* = 5145) and the preserved blood meals from *Mansonia* species (*n* = 144) collected using odour‐baited traps were also screened for *Plasmodium* spp. One *P. falciparum* parasite was found in the human blood meal obtained from a *M. africana* mosquito (Fig. 3). Moreover, *Plasmodiidae* species were amplified from 21 *An. obscurus* mosquitoes (0.4%) and one *An. paludis* mosquito (4.3%) (Fig. 3). Consistent with the lack of ape *Plasmodium* parasites in the chimpanzee population, phylogenetic analysis revealed that none of the *Plasmodiidae* species detected were of ape origin. Most of the *Plasmodiidae* sequences (*n* = 17) formed a single clade distinct from characterized *Plasmodium* species (Fig. 3). They did cluster with a *Plasmodium* spp. from an African buffalo (*Syncerus caffer*) from Gabon and a *Plasmodium* spp. from a marshbuck (*Tragelaphus spekii*) from Gabon (Bitome‐Essono *et al*., 2017). Three sequences (TC06, TC021 and TC019) clustered with a *Plasmodium* spp. that has previously been amplified from *An. moucheti* and *Anopheles marshalii* mosquitoes in Gabon (Boundenga *et al*., 2016). Two *Plasmodiidae* sequences (TC02 and TC08) clustered with a *Plasmodium* spp. from a *Coquillitidia* mosquito from Japan (Ejiri *et al*., 2008). In addition, these two *Plasmodiidae* sequences belong to a group of *Plasmodium* spp. that are found in birds (Ricklefs & Outlaw 2010).

## Discussion

When sampling mosquitoes at a chimpanzee rehabilitation site, situated within a natural chimpanzee habitat where wild mosquitoes would feed on the chimpanzees being rehabilitated, it was found that mosquito species exhibited different host preferences. Moreover, the location of the trap had a significant effect on the mosquito catches, as seen in other field studies on mosquito host preference (Hiscox *et al*., 2014; Pombi *et al*., 2014). Most of the mosquito species caught during the present study, including *An. obscurus* and *M. africana*, showed a generalistic host preference and were attracted to all of the host species tested. Interestingly, a *Culex* species belonging to the *Cx. decens* complex showed a much higher attraction towards chimpanzee odours than to human or cow odours. The blood‐feeding behaviour of mosquitoes is highly plastic and the adaptation of mosquitoes to available host species could have implications for pathogen transmission (Chaves *et al*., 2010; Takken & Verhulst 2013). Studies have shown that *Cx. decens* feeds on both birds and bats (Boreham & Snow 1973; Quan *et al*., 2010). Whether *Cx. decens* has developed a specialised host preference for chimpanzees remains to be investigated. The specialisation of mosquitoes could turn a generalist mosquito into a more dangerous anthropophilic vector of human infections, as seen for *Aedes aegypti*, which is now a dominant vector of Zika virus and dengue virus (Yakob *et al*., 2010; Scott & Takken 2012; Brown *et al*., 2014).

*Mansonia* mosquitoes are aggressive biting mosquitoes and are associated with Rift Valley fever virus, West Nile virus and *Wucheria bancrofti* (Fontenille *et al*., 1998; Diallo *et al*., 2005; Ughasi *et al*., 2012). The *Mansonia* species caught during the present study were all morphologically identified as *M. africana* and showed a strong preference for cow odour‐baited mosquito traps. Different studies in the past have observed different host preferences for *Mansonia* mosquitoes, ranging between anthropophilic, a high preference for cows and a generalistic host preference, respectively (Lefèvre *et al*., 2009; Busula *et al*., 2015; Omondi *et al*., 2015). Although the different results obtained could be a result of the study methodology, Busula *et al*. (2015) utilized a comparable experimental design to that used in the present study, indicating that, even within a mosquito species, different populations can have different host preferences (Takken & Verhulst 2013).

*Anopheles obscurus* was the predominant *Anopheles* species caught during the present study (97.4%). This typical marsh and swamp breeder is an opportunistic species biting both bovine and primate hosts (Boorman and Service, Boorman & Service 1960). *Anopheles obscurus* has also been associated with feeding on ungulate hosts such as *Cephalophus* (duikers) species and found to be infected with ungulate *Plasmodium* spp. (Boundenga *et al*., 2016; Makanga *et al*., 2017). Experimental studies have shown that *Anopheles* mosquitoes with different host preferences are similarly attracted towards non‐human primates and humans (Verhulst *et al*., 2018). Moreover, a field study by Makanga *et al*. (2016) identified mosquitoes biting both humans and great apes. The *Anopheles* mosquitoes in the present study were equally attracted towards chimpanzee and human odours. However, although 21 *An. obscurus* mosquitoes were infected with a variety of *Plasmodiidae* species, no primate *Plasmodium* was detected, which suggests that *An. obscurus* in not a competent vector. Because of the lack of well‐defined reference sequences, it was impossible to classify the parasites to genus level with any kind of certainty. Therefore, it was decided to assign them to the *Plasmodiidae* family, which includes both *Plasmodium* and *Hepatocystis*. Other mosquito species such as *An. gambiae*, *An. ziemanni*, *An. nili* and *An. moucheti* caught in the present study are known *Plasmodium* vectors and may play a role as bridge vectors in the circulation of *P. falciparum* in this area. Although their abundance was very low, this could still be sufficient to sustain transmission (Homan *et al*., 2016).

Human *P. falciparum* was found in a chimpanzee blood sample and a human blood sample from a *Mansonia* mosquito (Fig. 3), which indicated that chimpanzees can be infected with *P. falciparum*. Although a chimpanzee to chimpanzee transmission of *P. falciparum* cannot be ruled out, it is likely that this represents transmission of *P. falciparum* from humans to chimpanzees, which has previously been observed in apes held in captivity (Krief *et al*., 2010; Prugnolle *et al*., 2010; Pacheco *et al*., 2013; Ngoubangoye *et al*., 2016). By contrast, *P. falciparum* has never been detected in wild chimpanzees, bonobos or gorillas, which suggests that there is something unique about the sanctuary environment that facilitates cross‐species transmission. It is possible that the rangers working in the sanctuary harbour subclinical densities of *P. falciparum* (Rayner *et al*., 2011; Maselli *et al*., 2014; Rovira‐Vallbona *et al*., 2017), which, when picked up by anopheline mosquitoes, can be transmitted to the chimpanzees held in the sanctuary. However, it was not possible to identify bridge vectors for *P. falciparum* because none of the *Anopheles* specimens collected were infected with human *P. falciparum*, although some of the mosquitoes caught at Tchimpounga (*An. ziemanni*, *An. nili*, *An. moucheti* and *gambiae s.l*.) are known human malaria vectors (Greenwood *et al*., 2005; Scott & Takken 2012; Sinka *et al*., 2012). Additional work is needed to determine the factors that facilitate cross species transmission of *P. falciparum* in sanctuary apes.

Unravelling the trophic behaviour of mosquitoes in remote locations will provide us with valuable information on potential transmission pathways of vector‐borne diseases between non‐human primates. With increasing human activities in natural environments, the risk of zoonoses increases, as seen in the cases of a non‐human primate derived *P. vivax* infecting a Caucasian man travelling from Africa and the transmission of *Plasmodium knowlesi* from monkey to humans in South‐East Asia (Cox‐Singh *et al*., 2008; Singh *et al*., 2004; Paupy *et al*., 2013). This is further supported by recent zoonotic transmission of *P. simium* from simians to humans in the Atlantic Forest of Rio de Janeiro (Brasil *et al*., 2017). Mosquito plasticity in host preference has recently been shown as a major factor for disease outbreaks (Yakob *et al*., 2018). In addition, adaptation of arthropod‐borne viruses to new mosquito vectors is a major concern in the spread of vector‐borne diseases and has already caused outbreaks of chikungunya virus and West‐Nile virus (Kilpatrick *et al*., 2006, Kilpatrick *et al*., 2008, Tsetsarkin *et al*., 2007, 2016). The trophic behaviour of mosquitoes was determined in the present study using a variety of methods, including odour‐baited traps, analyses of blood meal and pathogens of mosquitoes. Each of these methods alone would have led to different conclusions, which showed that a combination of methods is required to be able to fully understand the behaviour of disease vectors. The present study provides evidence that the majority of mosquito species collected near wild apes are attracted to odours of multiple different species, including chimpanzees and humans, and may thus serve as bridge vectors for ape pathogens.

The identification of *P. falciparum* in both humans and chimpanzees suggests that there is active circulation of these parasites transmitted by anopheline mosquitoes, although it is unlikely that *An. obscurus* is part of this transmission cycle given that, regardless of their high abundance, no primate *Plasmodium* positive specimens were found. Primary human *Plasmodium* vectors such as *An. gambiae s.l*., *An. nili* and *An. moucheti*, were collected during this study period, which could have played a role as bridge vectors for the circulation of *P. falciparum*.

## Supporting information


**Figure S1**. Attraction of mosquito species to different host odours trapped with odour‐baited Suna traps.
**Table S1**. *Plasmodium* cytB sequences used in the phylogenetic analyses.
**Table S2**. Samples collected and prevalence of *Plasmodium*.
**Table S3**. Pairwise comparisons of mosquito trap catches with different odour baits.
