# Supplementary material for: Attraction of mosquitoes to primate odours and implications for zoonotic Plasmodium transmission
Source: Med Vet Entomol. 2019 Aug 17;34(1):17–26. doi: 10.1111/mve.12402 (PMC7002228; doi:10.1111/mve.12402)
Supplement: Supplementary file 1 — Figure S1. Attraction of mosquito species to different host odours trapped with odour‐baited Suna traps. Table S1. Plasmodium cytB sequences used in the phylogenetic analyses. Table S2. Samples collected and prevalence of Plasmodium. Table S3. Pairwise comparisons of mosquito trap catches with different odour baits. [file MVE-34-17-s001.pdf]

Supplementary material

Attraction of mosquitoes to primate host odours and implications for zoonotic *Plasmodium* transmission

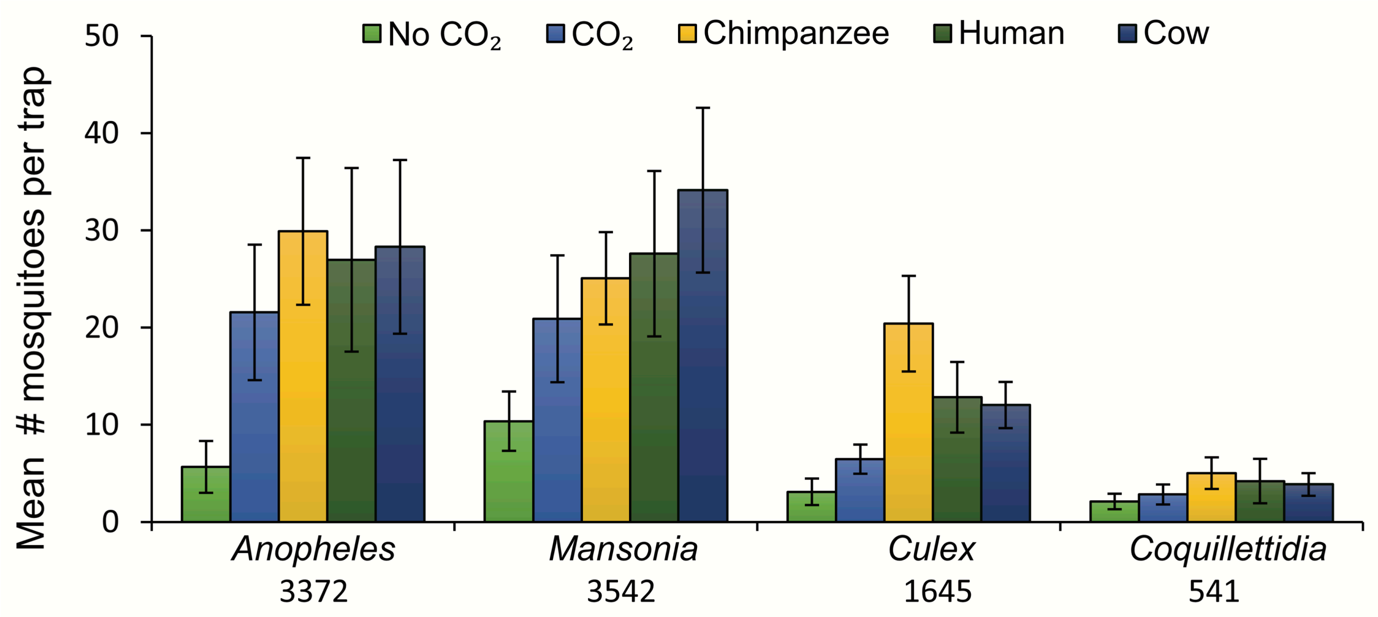

Figure S1. Attraction of mosquito species to different host odours trapped with odour baited Suna traps. Bars represent the mean number of mosquitoes trapped per odour baited Suna trap per night using different host odour treatments: no odours, CO_2 only, chimpanzee odours with CO_2, human odours with CO_2 and cow odours with CO_2. Error bars represent standard error of the mean. Numbers in the bars indicate the total number of mosquitoes caught per species. N = 30 trapping nights.

Table S1. Samples collected and Plasmodium prevalence.

Host species	Type of sample	Number of samples	*Plasmodium* sp. detected
Pan troglodytes	Blood	85	2 (2.4 %)
Mosquito	Whole specimen		
Aedes spp.		68	n/a
Mansonia spp.		6097	n/a
Culex spp.		1742	n/a
Coquillettidia spp.		549	n/a
Anopheles spp.		5145	22 (0.4 %)
An. obscurus		5002	21 (0.4 %)
An. ziemanni		87	0
An. paludis		23	1 (4.3 %)
An. gambiae s.l.		21	0
An. nili		6	0
An. moucheti		4	0
An. spp.		2	0
Mansonia	Blood meal (human)	244	1 (0.4 %)

Table S2. Pairwise comparisons of mosquito trap catches with different odour baits.

P-values (GLM) shown are based on proportions of caught mosquitoes using the Suna trap with different odour baits. Treatments are significant different at P <0 .05. Mean number of trapped mosquitoes can be found in Table 1.

Treatments		*Anopheles spp.*	*Mansonia spp.*	*Culex spp.*	*Coquillettidia spp.*
Chimpanzee + CO_2	CO_2	0.031	0.311	< 0.001	0.270
	Cow + CO_2	0.577	0.022	0.002	0.348
	Human+ CO_2	0.240	0.831	0.002	0.326
	No CO_2	< 0.001	<0.001	< 0.001	0.115
CO_2	Chimpanzee + CO_2	0.031	0.311	< 0.001	0.270
	Cow + CO_2	0.102	0.001	0.002	0.825
	Human + CO_2	0.306	0.060	0.002	0.867
	No CO_2	< 0.001	< 0.001	0.083	0.615
Cow + CO_2	Chimpanzee + CO_2	0.577	0.022	0.002	0.348
	CO_2	0.102	0.001	0.002	0.825
	Human + CO_2	0.549	0.036	0.977	0.957
	No CO_2	< 0.001	<0.001	< 0.001	0.456
Human + CO_2	Chimpanzee + CO_2	0.240	0.831	0.002	0.326
	CO_2	0.306	0.060	0.002	0.867
	Cow + CO_2	0.276	0.036	0.977	0.957
	No CO_2	< 0.001	<0.001	< 0.001	0.492
No CO_2	Chimpanzee + CO_2	< 0.001	<0.001	< 0.001	0.115
	CO_2	< 0.001	<0.001	0.083	0.615
	Cow + CO_2	< 0.001	<0.001	< 0.001	0.456
	Human + CO_2	< 0.001	<0.001	< 0.001	0.492

Table S3. ***Plasmodium*** **Cyt-b sequences used in phylogenetic analyses**

Species	Accession number
TC01_*An. obscurus*	MK502145
TC02_*An. obscurus*	MK502146
TC03_*An. obscurus*	MK502147
TC04_*An. obscurus*	MK502148
TC05_*An. obscurus*	MK502149
TC06_*An. obscurus*	MK502150
TC07_*An. obscurus*	MK502151
TC08_*An. obscurus*	MK502152
TC09_*An. obscurus*	MK502153
TC10_*An. obscurus*	MK502154
TC11_*An. obscurus*	MK502155
TC12_*An. obscurus*	MK502156
TC13_*An. obscurus*	MK502157
TC14_*An. obscurus*	MK502158
TC15_*An. obscurus*	MK502159
TC16_*An. obscurus*	MK502160
TC17_*An. obscurus*	MK502161
TC18_*An. obscurus*	MK502162
TC19_*An. obscurus*	MK502163
TC20_*An. paludis*	MK502164
TC21_*An. obscurus*	MK502165
TC22_*An. obscurus*	MK502166
Plasmodium sp.	KY631955
Plasmodium sp.	KY631942
Plasmodium sp.	AB308052
P. odocoilei	MH177860
Plasmodium sp._An.vinckei	KU318100
Plasmodium sp._An.obscurus	KU318108
Plasmodium sp._An.obscurus	KU318107
Plasmodium sp._An.moucheti	KU318051
Plasmodium sp._An.marshallii	KU318048
Plasmodium sp._An.gabonensis	KU318037
P. minuoviride	EU834703
P. lomamiensis	KY790541
P. hylobati	AF069618
P. vivax	NC7243
P. relictum	AY733088
P. relictum	AY099032
P. reichenowi	AJ251941
P. preafalciparum	HM235296
P. ovale	AB354571
P. mexicanum	AB375765
P. malariae	AB354570
P. knowlesi	AY722797
P. juxtanucleare	AB250415
P. gonderi	AB434918
P. giganteum	AY099053
P. gallinaceum	AB250690
P. gaboni	FJ895307
P. falciparum	AY282924
P. elongatum	AF069611
P. cynomolgi	AB434919
P. chabaudi	AY099050
P. blacklocki	HM235304
P. billcollinsi	GQ355477
P. azurophilum	AY099055
P. athururi	AY099054
P. agamae	AY099048
P. adleri	HM235303
Hepatocystis sp.	JQ070892
Hepatocystis sp.	JQ070884
Hepatocystis sp.	JQ070873
Hepatocystis sp.	JF923759
Hepatocystis sp.	DQ396528
Hepatocystis sp.	AF069626
Haemosporida sp.	KT367837
